# Predictors of timely linkage‐to‐ART within universal test and treat in the HPTN 071 (PopART) trial in Zambia and South Africa: findings from a nested case‐control study

**DOI:** 10.1002/jia2.25037

**Published:** 2017-12-18

**Authors:** Kalpana Sabapathy, Constance Mubekapi‐Musadaidzwa, Chama Mulubwa, Ab Schaap, Graeme Hoddinott, Anne Stangl, Sian Floyd, Helen Ayles, Sarah Fidler, Richard Hayes, for the HPTN 071 (PopART) study team

**Affiliations:** ^1^ London School of Hygiene and Tropical Medicine London United Kingdom; ^2^ Desmond Tutu TB Centre Western Cape South Africa; ^3^ Desmond Tutu TB Centre Department of Paediatrics and Child Health Faculty of Medicine and Health Sciences Stellenbosch University Cape Town South Africa; ^4^ International Centre for Research on Women Washington DC USA; ^5^ Imperial College London London United Kingdom

**Keywords:** linkage to care, ART initiation, cascade of care, differentiated care, universal test and treat, universal treatment, immediate ART, treatment as prevention

## Abstract

**Introduction:**

HPTN 071 (PopART) is a three‐arm community randomized trial in Zambia and South Africa evaluating the impact of a combination HIV prevention package, including universal test and treat (UTT), on HIV incidence. This nested study examined factors associated with timely linkage‐to‐care and ART initiation (TLA) (i.e. within six‐months of referral) in the context of UTT within the intervention communities of the HPTN 071 (PopART) trial.

**Methods:**

Of the 7572 individuals identified as persons living with HIV (PLWH) (and not on antiretroviral treatment (ART)) during the first year of the PopART intervention provided by Community HIV‐care Providers (CHiPs) through door‐to‐door household visits, individuals who achieved TLA (controls) and those who did not (cases), stratified by gender and community, were randomly selected to be re‐contacted for interview. Standardized questionnaires were administered to explore factors potentially associated with TLA, including demographic and behavioural characteristics, and participants’ opinions on HIV and related services. Odds ratios comparing cases and controls were estimated using a multi‐variable logistic regression.

**Results:**

Data from 705 participants (333 cases/372 controls) were analysed. There were negligible differences between cases and controls by demographic characteristics including age, marital or socio‐economic position. Prior familiarity with the CHiPs encouraged TLA (aOR of being a case: 0.58, 95% CI: 0.39 to 0.86, *p* = 0.006).

Participants who found clinics overcrowded (aOR: 1.51, 95% CI: 1.08 to 2.12, *p* = 0.006) or opening hours inconvenient (aOR: 1.63, 95% CI: 1.06 to 2.51, *p* = 0.02) were less likely to achieve TLA, as were those expressing stronger feelings of shame about having HIV (*p*_trend_ = 0.007). Expressing “not feeling ready” (aOR: 2.75, 95% CI: 1.89 to 4.01, *p* < 0.001) and preferring to wait until they felt sick (aOR: 2.00, 95% CI: 1.27 to 3.14, *p* = 0.02) were similarly indicative of being a case. Worrying about being seen in the clinic or about how staff treated patients was not associated with TLA.

While the association was not strong, we found that the greater the number of self‐reported lifetime sexual partners the more likely participants were to achieve TLA (*p*_trend_ = 0.06). There was some evidence that participants with HIV‐positive partners on ART were less likely to be cases (aOR: 0.75, 95% CI: 0.53 to 1.06, *p* = 0.07).

**Discussion:**

The lack of socio‐demographic differences between cases and controls is encouraging for a “universal” intervention that seeks to ensure high coverage across whole communities. Making clinics more “patient‐friendly” could enhance treatment uptake further. The finding that those with higher risk behaviour are more actively engaging with UTT holds promise for treatment‐as‐prevention.

## 1 Introduction

The concept of universal test and treat (UTT) has been widely promoted for approximately the last decade, and definitive evidence for the efficacy of treatment as prevention at the level of individual partnerships has been available since the results of the HPTN (HIV Prevention Trials Network) 052 trial were announced in 2011 1, 2. Other studies have since provided clear evidence on the benefits of early treatment for the health of the person living with HIV (PLWH) 3, 4. The World Health Organization (WHO) has revised its guidelines, to recommend that all PLWH be offered antiretroviral treatment (ART) irrespective of CD4 count, also known as “immediate treatment” 5.

**Figure 1:**
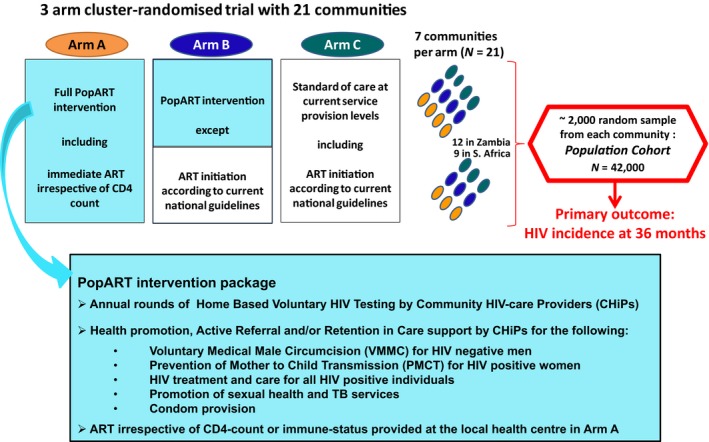
Overview of the HPTN 071 (PopART) trial.

The HPTN 071 (Population Effects of Antiretroviral Therapy to Reduce HIV Transmission (PopART)) trial has been underway in 21 communities in Zambia and South Africa to examine the impact of a combination prevention package including UTT on HIV‐incidence at a community level. The trial consists of three arms as described elsewhere 6 and illustrated in Figure 1, and the full intervention arm (Arm A) has offered UTT from the launch of the trial in 2014. Data from the first year of delivering the intervention in Zambia indicate that among PLWH an estimated 78% of men and 87% of women were aware of their HIV‐positive status and approximately three‐quarters of them were on ART after one year 7.

In order for UTT to have maximal impact in reducing community‐level HIV‐incidence, uptake of treatment would have to be wide‐spread across subsets of the population with different socio‐demographic and behavioural characteristics. There is evidence on factors associated with initiation of ART, usually among individuals who have already linked‐to‐care at least once since HIV diagnosis and from contexts where ART eligibility criteria applied 8, 9, 10, 11, 12, 13. More scarce are data from settings providing immediate treatment or UTT, with the latter not only making ART immediately available for all who have already presented to health‐facilities, but first seeking to test everyone in the community (universal testing) 14. Although a UTT approach substantially simplifies treatment by removing ART initiation criteria, providers must still negotiate all the steps along the cascade of care, to link clients to care following HIV detection in the community and achieve timely initiation of ART. Evidence from the ANRS TasP trial indicates that linkage‐to‐care is a major barrier to achieving high treatment coverage 15. UTT also involves attempting to initiate treatment in a timely fashion, in individuals who were diagnosed as part of a universal testing programme in the community, who may not otherwise have sought to find out their HIV status. While this helps achieve universal knowledge of HIV status and identify as many PLWH as possible, it may also pose challenges for treatment readiness in addition to other potential barriers.

The study described here examined the factors associated with the uptake of universal treatment, specifically timely linkage‐to‐care and initiation of treatment (which we will refer to as timely linkage‐to‐ART (TLA)) following door‐to‐door universal testing, during the first year of the PopART UTT intervention. The study objectives were to identify differences between those who had not achieved TLA (cases) and those who had done so (controls). TLA was defined as linkage‐to‐care and initiation of ART within six months of referral by Community HIV‐care Providers (CHiPs).

## 2 Methods

The nested case‐control study was carried out in PopART Arm A communities which offered UTT: four in Zambia and three in South Africa (Figure 1). CHiPs (lay counsellors employed and trained to work in their own communities), provided a door‐to‐door community‐based package of services (Figure 1) and captured the details of all individuals who consented to the PopART intervention on an electronic register 6. During the initial CHiP home visit, HIV was detected through HIV testing and counselling (HTC) or by individuals self‐reporting HIV‐positive status. All PLWH who were not on ART were offered referral to the local health‐facility for linkage‐to‐ART. Follow‐up home visits were conducted to ascertain linkage and ART initiation outcomes and recorded in the CHiP electronic register.

Community members who were referred between January 2014 and January 2015, during the first year of the PopART intervention and had had at least six months to achieve TLA (by July 2015) were eligible for the case‐control study. Furthermore, participants had to be ≥18 years old and be able and willing to provide informed consent.

The data on TLA entered into the database at a follow‐up home visit by CHiPs, more than six months after referral (to allow time for TLA), were used as a starting point for random selection of study participants. Participants who had achieved TLA within six months of referral by CHiPs were eligible to be controls; individuals who had been followed up after six months but had not started ART at all or only started after six months were potential cases. From all those who were eligible to be cases in the database, approximately 140 potential cases per community (50% men and 50% women) were randomly selected. These were placed in a random order (stratified by community and gender), on a sequential list of potential subjects. An equal number of gender‐matched individuals per community, who did manage TLA (controls) were then randomly selected in the same way. A sample in excess of the number required for recruitment (approximately 60 cases and 60 controls per community, stratified by gender) was selected in anticipation of difficulties in finding participants, given the mobility and migration in the study communities. Due to a wide range in community sizes, the numbers of individuals meeting the case and control definition varied across communities and some communities had fewer individuals than required by the study while others had much larger numbers. This meant that in the smaller communities everyone who had been referred was selected, rather than a random subset.

Verbal permission to allow case‐control (CC) field research assistants (RAs) to approach participants was obtained by the CHiP staff who had previously referred individual community members for TLA. Written informed consent for study participation was then obtained by CC study RAs. RAs conducted electronically administered surveys with standardized questionnaires. Questionnaire themes were informed by current evidence in the literature and anecdotal local information on factors that may influence TLA. Participants’ case or control status was not given to RAs.

The final sample size of approximately 700 participants (1:1 case:control ratio) provided just over 80% study power to detect associations with odds ratios of approximately 1.75 or higher (or approximately 0.5 or lower), for explanatory variables with 15% prevalence in controls (*α* = 0.05). Odds ratios were estimated using a multivariable logistic regression and all models included community and gender to account for the frequency‐matched sampling strategy. Age category was then included as an *a priori* potentially confounding variable. Additional variables which showed at least weak evidence of association with TLA were included as appropriate. Likelihood ratio testing (LRT) was done to assess the statistical evidence of association. Evidence of interaction with gender and country was explored. For variables with three or more response categories and potential for a dose‐response relationship, tests for trend were performed.

The study was approved by the ethics committees of the University of Zambia, Stellenbosch University and London School of Hygiene and Tropical Medicine.

## 3 Results

### 3.1 Demographic, sexual behaviour and health‐related characteristics

During the first year of the PopART intervention 152,383 individuals consented to participate in the PopART intervention in the 7 arm A communities. Of these, 11% (n = 16,112 individuals) were identified as living with HIV (newly diagnosed or self‐report of known HIV‐positive status) and 7572 were referred to HIV care, among those not on ART at the time of referral. After allowing at least six months for TLA, 68% (n = 5161) were documented as either having started ART within six months after referral, or were followed up >6 months after referral and had not started ART. Of these, 47% (n = 2444 individuals) had linked‐to‐care and initiated ART within six months of referral and 53% (n = 2717) had not.

**Figure 2:**
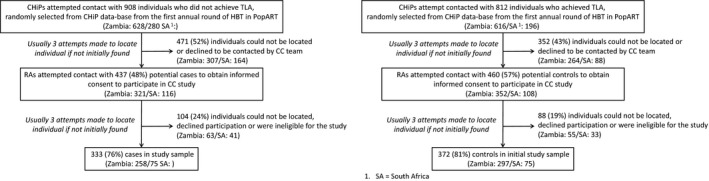
Case and control selection process.

Of the 2717 potential cases who did not achieve TLA, 908 were randomly selected to be contacted by CHiPs, and 437 (48%) were found and agreed to be contacted by the CC field RAs. Of them, 333 (76%) cases consented to participate in the study (Figure 2a). Of the 812 potential controls who achieved TLA and were randomly selected, 460 (57%) agreed to be contacted by the CC RAs and a slightly greater proportion were successfully recruited (372 (81%)) (Figure 2b).

Data from 705 participants were included in the final analysis: 79% (555) from four communities in Zambia and 21% (150) from three communities in South Africa. There were fewer men (approximately 40%) than women (approximately 60%) in the study, but proportions were balanced by case/control status (Table 1). When compared with the randomly selected sample, there was a lower proportion of 18 to 24 year olds in the final study population (10% in the final study sample vs. 18% in the randomly selected sample of both cases and controls) and a higher proportion of ≥45 year olds (22% vs. 11% among cases, and 25% vs. 15% among controls).

**Table 1:** Demographic, sexual behaviour and health‐related characteristics of cases and controls

	Controls (achieved TLA) N (%)	Cases (did not achieve TLA) N (%)	Odds ratio^a^	LRT^b^ *p‐value*, 95% confidence interval	Adjusted odds ratio^c^	LRT^b^ *p*‐value, 95% confidence interval
Total						
Gender^d^						
Male	151 (41)	132 (40)				
Female	220 (59)	201 (60)				
Demographic and socio‐economic characteristics						
Age category						
18 to 24 years	36 (10)	32 (10)	1	*0.86*	1	*0.60*
25 to 34 years	129 (35)	118 (35)	1.04	0.60 to 1.80	1.05	0.59 to 1.89
35 to 44 years	115 (31)	110 (33)	1.08	0.61 to 1.89	1.08	0.60 to 1.95
≥45 years	92 (25)	73 (22)	0.91	0.50 to 1.63	0.82	0.45 to 1.52
Marital status						
Never married	48 (13)	55 (16)	1	*0.48*	1	*0.27*
Currently married	215 (58)	184 (55)	0.75	0.46 to 1.21	0.77	0.45 to 1.32
Previously married^e^	109 (29)	94 (28)	0.75	0.44 to 1.28	0.63	0.35 to 1.12
Educational attainment						*p*_trend_ 0.11^f^
Primary (Grade 0 to 7)	183 (49)	153 (46)	1	*0.76*	1	*0.21*
Junior secondary (Grade 8 to 9)	94 (25)	84 (25)	1.09	0.75 to 1.58	1.15	0.77 to 1.72
Senior secondary (Grade 10 to 12)/higher education	94 (25)	96 (29)	1.22	0.83 to 1.78	1.45	0.96 to 2.19
Employment						
None	181 (49)	166 (50)	1	*0.42*	1	*0.41*
Casual/seasonal/occasional	61 (16)	60 (18)	1.12	0.71 to 1.75	1.18	0.74 to 1.89
Self employed	67 (18)	64 (19)	1.12	0.73 to 1.73	1.22	0.77 to 1.92
Formal wage	63 (17)	43 (13)	0.75	0.47 to 1.19	0.79	0.49 to 1.29
SES (PCA^g^ of HH factors & assets^h^)						
Lower	181 (49)	176 (53)	1	*0.27*	1	*0.43*
Higher	191 (51)	157 (47)	0.84	0.61 to 1.15	0.87	0.63 to 1.22
Sexual behaviour						
No of lifetime sexual partners						*p*_trend_ 0.06^f^
1	33 (9)	47 (14)	**1**	***0.02***	**1**	***0.04***
2 to 5	221 (60)	192 (58)	**0.57**	**0.34 to 0.93**	**0.55**	**0.33 to 0.92**
6 to 9	49 (13)	48 (14)	0.59	0.30 to 1.13	0.56	0.28 to 1.10
≥10	68 (18)	45 (14)	**0.40**	**0.21 to 0.75**	**0.38**	**0.19 to 0.74**
Self‐assessment of sexual risk (“My sexual behaviour (incl. partner(s) I have had), has put me at risk of getting HIV”)						
Low	180 (49)	181 (55)	1	*0.08*	1	*0.13*
High	191 (51)	151 (45)	0.75	0.55 to 1.03	0.77	0.55 to 1.08
HIV and health factors						
Has HIV status been disclosed to anyone						
N	38 (10)	74 (22)	**1**	***<0.001***	**1**	***<0.001***
Y	334 (90)	259 (78)	**0.39**	**0.25 to 0.59**	**0.39**	**0.25 to 0.60**
HIV & ART status of spouse/main partner^i^ (in last 12 m)						
No sexual partner in last 12 m/partner HIV status not known or reported HIV negative	214 (58)	204 (61)	1	***0.002***	1	***0.005***
HIV positive partner not on ART	20 (5)	37 (11)	**1.96**	**1.10 to 3.49**	**2.02**	**1.11 to 3.66**
HIV positive partner on ART	138 (37)	92 (28)	**0.70**	**0.50 to 0.98**	0.72	0.51 to 1.03
Had participant linked to care prior to referral by CHiP						
Not linked prior to CHiP	280 (75)	274 (82)	1	***0.04***	1	*0.09*
Linked prior to CHiP but not in care when referred	29 (8)	21 (6)	0.70	0.38 to 1.28	0.79	0.42 to 1.47
Linked prior to CHiP and in care when referred (not self‐reported on ART)	63 (17)	38 (11)	**0.59**	**0.38 to 0.92**	**0.60**	**0.38 to 0.96**
Most recent CD4‐count						
0 to 350	60 (16)	34 (10)	**1**	***<0.001***	**1**	***<0.001***
351 to 500	44 (12)	18 (5)	0.72	0.36 to 1.46	0.69	0.33 to 1.43
≥501	53 (14)	34 (10)	1.17	0.63 to 2.16	1.17	0.62 to 2.20
Not done/don't know CD4‐count	215 (58)	247 (74)	**2.21**	**1.37 to 3.58**	**1.94**	**1.18 to 3.21**
Health in last 12 m						
Not been unwell	269 (72)	225 (68)	1	*0.33*	1	*0.18*
Unwell, not hospitalized	63 (17)	71 (21)	1.35	0.91 to 2.04	1.46	0.97 to 2.21
Unwell and hospitalized	40 (11)	37 (11)	1.07	0.66 to 1.74	1.19	0.72 to 1.99
AUDIT score						
Audit score ≤7	253 (68)	226 (68)	1	*0.85*	1	*0.73*
Audit score ≥8	119 (32)	107 (32)	1.03	0.73 to 1.45	1.07	0.74 to 1.54

Values in italics are *p*‐values, values in bold (with or without italics) indicate statistically significant values.

a *A priori* adjusted for gender and community to reflect sampling strategy.

b Likelihood ratio test.

c Multivariable model *a priori* including gender, community, age category as well as demographic/behavioural factors which were associated with case/control status (i.e. whether CHiP was known to participant prior to PopART to home‐visit, whether HIV status has been disclosed, whether partner is HIV positive and on ART, and lifetime number of sexual partners).

d One participant (control) with missing gender data.

e Previously married = separated/divorced/widowed.

f *p*‐value for test for tend.

g Principal components analysis.

h HH factors detailed house structure, water, sanitation, electricity and cooking fuel used; assets listed were: working cell‐phone, bicycle, motorcycle or scooter, car/bakkie, electricity to house, television set, fridge/freezer, radio, computer/laptop, CD or MP3 player, stereo/cassette/other music player, “none of the above.”

i Participant's own definition of “main partner.”

Most participants were married, had had primary education only and were unemployed (2% of all participants were students). There were no differences by case/control status for any of these demographic characteristics, overall.

The majority of participants reported two to five lifetime sexual partners and the number of partners was associated with case/control status. There is a suggestion of a trend that the greater the number of partners the more likely participants were to achieve TLA (with diminishing odds ratios (aORs) of being a case, *p*_trend_ = 0.06) (Table 1).

The HIV and ART status of the participant's main partner (of the last 12 months) was associated with case/control status. Compared to those who did not report having a known HIV‐positive partner, participants who had HIV‐positive partners not on ART (although there were relatively few in that category) were twice as likely to be cases themselves (aOR: 2.02, 95% CI: 1.11 to 3.66, *p* = 0.02). Participants whose HIV‐positive partners were on ART were less likely to be cases (OR: 0.70, 95% CI: 0.50 to 0.98, *p* = 0.04), but the evidence of association was weaker in the adjusted model (aOR: 0.75, 95% CI: 0.53 to 1.06, *p* = 0.07).

Participants who had previously linked‐to‐care (prior to CHiP referral) and were still in care (but not on ART) when the initial CHiP home visit was conducted were less likely to be cases than those who had never previously linked (aOR: 0.60, 95% CI: 0.38 to 0.96, *p* = 0.03). Although there were relatively few participants who reported that they had previously linked but had defaulted from care, when compared against those who had never linked there was no evidence of a difference (aOR: 0.79, 95% CI: 0.42 to 1.47, *p* = 0.45) (Table 1). There was no relationship between latest CD4‐count (self‐reported at the time of the CC study by those who had had it done) and case/control status. However, not having had a CD4‐count done or not knowing one's result was predictive of being a case (aOR: 1.94, 95% CI: 1.18 to 3.21, *p* = 0.009) when compared with those who had a CD4‐count which was low (0 to 350/cm^3^).

### 3.2 Participants’ perceptions of HIV service factors which may affect TLA

Cases and controls were asked (identical) standardized questions about factors that encourage TLA (regardless of whether TLA was achieved). Prior familiarity with the CHiP who delivered the intervention encouraged TLA (aOR of being a case: 0.58, 95% CI: 0.39 to 0.86, *p* = 0.006) (Table 2). The majority of participants accepted HIV testing with the CHiP, and this appears to be associated with being a case, although not reaching statistical significance in the adjusted model (aOR: 1.60, 95% CI: 0.95 to 2.70, *p* = 0.07) when compared with individuals who self‐reported HIV‐positive status.

**Table 2:** Participants perceptions of HIV service factors which may affect initiation of timely treatment

	Controls (achieved TLA) N (%)	Cases (did not achieve TLA) N (%)	Odds ratio^a^	LRT^b^ *p*‐value, 95% confidence interval	Adjusted odds ratio^c^	LRT^b^ *p*‐value, 95% confidence interval
CHiP related factors						
Was the CHiP previously known to participant (prior to PopART home‐visit)						
N	259 (70)	253 (76)	**1**	***0.05***	**1**	***0.006***
Y	113 (30)	80 (24)	**0.70**	**0.48 to 1.01**	**0.58**	**0.39 to 0.86**
Did participant have an HIV test with CHiP^d^						
N	47 (13)	27 (8)	1	*0.05*	1	*0.07*
Y	325 (87)	306 (92)	1.63	0.99 to 2.71	1.60	0.95 to 2.70
Was the CHiP someone you could talk to openly?						*p*_trend_ 0.01^e^
Strongly disagree/Disagree	7 (2)	14 (4)	1	***0.02***	1	***0.04***
Agree	66 (18)	80 (24)	0.59	0.22 to 1.58	0.64	0.23 to 1.81
Strongly agree	299 (80)	239 (72)	**0.37**	**0.14 to 0.97**	0.41	0.15 to 1.13
Factors specifically affecting linkage to care						
Time constraints affecting linkage to care						
Already in care/time not a constraint for LTC	289 (78)	231 (69)	**1**	***0.02***	**1**	***0.02***
Time constraints due to livelihood/housework or both	82 (22)	102 (31)	**1.62**	**1.14 to 2.30**	**1.64**	**1.14 to 2.38**
Among those never previously registered for care prior to CHiP referral (N = 603), did the following affect linkage to care?						
“Clinic is only open when I am at work”						
N	262 (85)	231 (78)	**1**	***0.03***	**1**	***0.02***
Y	46 (15)	64 (22)	**1.63**	**1.06 to 2.51**	**1.63**	**1.06 to 2.51**
“Clinic is too crowded”						
N	181 (59)	144 (49)	**1**	***0.12***	**1**	***0.006***
Y	127 (41)	151 (51)	**1.50**	**1.07 to 2.11**	**1.51**	**1.08 to 2.12**
“I could not go to the clinic because it is too far away/because of the time it would take to travel there”						
N	299 (97)	273 (93)	**1**	***0.02***	**1**	***0.03***
Y	9 (3)	22 (7)	**2.52**	**1.13 to 5.61**	**2.55**	**1.14 to 5.69**
“I am not ready to go the clinic for HIV care”						
N	249 (81)	183 (62)	**1**	***<0.001***	**1**	***<0.001***
Y	59 (19)	112 (38)	**2.68**	**1.84 to 3.89**	**2.75**	**1.89 to 4.01**
“I will only go if/when I feel sick”						
N	270 (88)	232 (79)	**1**	***0.003***	**1**	**0.02**
Y	38 (12)	63 (21)	**1.96**	**1.26 to 3.07**	**2.00**	**1.27 to 3.14**

Values in italics are *p*‐values, values in bold (with or without italics) indicate statistically significant values.

a *A priori* adjusted for gender and community to reflect sampling strategy.

b Likelihood ratio test.

c Multivariable model *a priori* including gender, community, age category as well as demographic/behavioural factors which were associated with case/control status (i.e. whether CHiP was known to participant prior to PopART to home‐visit, whether HIV status has been disclosed, whether partner is HIV positive and on ART, and lifetime number of sexual partners).

d Participants who did not have a test with CHiPs were those who self‐reported known HIV‐positive status, while those who had an HIV test with CHiPs were likely to be previously undiagnosed.

e *p*‐value for test for tend.

Participants who reported time constraints due to work or housework as a factor which discouraged linkage‐to‐care were more likely to be cases (aOR: 1.64, 95% CI: 1.14 to 2.38, *p* = 0.02). When restricted to those who had not previously linked‐to‐care prior to the CHiP referral (N = 603), a number of factors related to accessing the clinic, including inconvenient clinic opening hours (aOR: 1.63, 95% CI: 1.06 to 2.51, *p* = 0.02); overcrowding in the clinic (aOR: 1.51, 95% CI: 1.08 to 2.12, *p* = 0.006); and distance/time to travel to clinic (aOR: 2.55, 95% CI: 1.14 to 5.69, *p* = 0.009) were associated with being a case (although very few complained of the latter in the urban and peri‐urban settings that characterize all our communities) (Table 2). Expressing “not feeling ready” (aOR: 2.75, 95% CI: 1.89 to 4.01, *p* < 0.001) and preferring to wait until they felt sick (aOR: 2.00, 95% CI: 1.27 to 3.14, *p* = 0.02) were similarly indicative of being a case. Neither being worried about being seen in the clinic or perceptions about how staff treated patients was associated with TLA (data not shown).

### 3.3 Perceived advantages and disadvantages to the individual of achieving TLA (including stigmatizing attitudes)

Participants who said that they would start ART for their own health, even without feeling unwell, were more likely to achieve TLA (aOR of being a case: 0.53, 95% CI: 0.38 to 0.75, *p* < 0.001) as were those who said they would comply with CHiPs/clinic staff's advice to link‐to‐ART without delay (aOR of being a case: 0.56, 95% CI: 0.34 to 0.91, *p* = 0.02). There was weak evidence that knowing others who were well on ART (aOR: 0.74, 95% CI: 0.53 to 1.03, *p* = 0.08) encouraged TLA. There was no strong association between case/control status and stating that protecting a partner from acquiring HIV was a factor which encouraged TLA (aOR: 0.81, 95% CI: 0.58 to 1.12, *p* = 0.19) (Table 3).

**Table 3:** Participants’ perceptions of advantages and disadvantages of initiation of timely treatment

	Controls (achieved TLA) N (%)	Cases (did not achieve TLA) N (%)	Odds ratio^a^	LRT^b^ *p*‐value, 95% confidence interval	Adjusted odds ratio^c^	LRT^b^ *p*‐value, 95% confidence interval
Individual level factors encouraging initiation of timely treatment (“Did any of the following encourage you start ART?”)						
“For my health even though I don't feel unwell”						
N	96 (26)	127 (38)	**1**	***<0.001***	**1**	***<0.001***
Y	275 (74)	206 (62)	**0.54**	**0.39 to 0.75**	**0.53**	**0.38 to 0.75**
Recommended by HCW (CHiP/clinic staff)						
N	35 (9)	55 (17)	**1**	***0.005***	**1**	***0.02***
Y	336 (91)	278 (83)	**0.52**	**0.33 to 0.82**	**0.56**	**0.34 to 0.91**
To protect partner from getting HIV						
N	189 (51)	188 (56)	1	0.11	1	0.19
Y	182 (49)	145 (44)	0.78	0.58 to 1.06	0.81	0.58 to 1.12
“I know someone/others who are well on ART and want to be on it too”						
N	142 (38)	155 (47)	**1**	***0.01***	1	*0.08*
Y	229 (62)	178 (53)	**0.67**	**0.48 to 0.92**	0.74	0.53 to 1.03
Individual level factors discouraging initiation of timely treatment (“Did any of the following discourage you from starting ART?”)						
“I was worried someone would find out about my HIV because of taking treatment/going to the clinic”						
N	278 (75)	232 (70)	1	*0.1*	1	*0.15*
Y	93 (25)	101 (30)	1.33	0.95 to 1.86	1.30	0.91 to 1.87
“I was/am not ready to take ART”						
N	285 (77)	196 (59)	**1**	***<0.001***	**1**	***<0.001***
Y	86 (23)	137 (41)	**2.42**	**1.73 to 3.38**	**2.25**	**1.58 to 3.21**
“I don't think the treatment works so there is no point in starting”						
N	333 (90)	285 (86)	1	*0.12*	1	*0.16*
Y	38 (10)	48 (14)	1.44	0.91 to 2.29	1.42	0.87 to 2.30
“I don't like the idea of taking life‐long treatment”						
N	288 (78)	234 (70)	**1**	***0.03***	**1**	***0.05***
Y	83 (22)	99 (30)	**1.48**	**1.04 to 2.09**	**1.44**	**1.00 to 2.07**
Stigmatizing attitudes which may affect initiation of timely treatment						
“People living with or thought to be living with HIV are verbally insulted, harassed and/or threatened”						*p*_trend_ 0.27^d^
Strongly disagree	46 (12)	34 (10)	1	*0.17*	1	*0.08*
Disagree	76 (20)	83 (25)	1.47	0.85 to 2.53	1.64	0.92 to 2.92
Agree	137 (37)	133 (40)	1.28	0.76 to 2.15	1.30	0.76 to 2.23
Strongly agree	113 (30)	83 (25)	0.95	0.55 to 1.63	0.95	0.54 to 1.66
“I have felt ashamed because of my HIV status”						*p*_trend_ 0.007^d^
Strongly disagree	118 (32)	86 (26)	1	*0.02*	1	***0.05***
Disagree	139 (37)	106 (32)	1.10	0.74 to 1.64	1.20	0.78 to 1.84
Agree	59 (16)	74 (22)	**1.83**	**1.12 to 2.98**	**1.82**	**1.10 to 3.03**
Strongly agree	56 (15)	67 (20)	**1.70**	**1.06 to 2.72**	**1.71**	**1.05 to 2.79**
“I have been excluded from social gatherings or activities because I have HIV”						*p*_trend_ 0.16^d^
Strongly disagree	162 (44)	134 (40)	1	*0.73*	1	*0.30*
Disagree	187 (50)	177 (53)	1.14	0.82 to 1.59	1.31	0.94 to 1.89
Strongly agree/agree	23 (6)	22 (7)	1.13	0.59 to 2.16	1.40	0.71 to 2.76

ART, antiretroviral treatment; LRT, likelihood ratio testing.

Values in italics are *p*‐values, values in bold (with or without italics) indicate statistically significant values.

a *A priori* adjusted for gender and community to reflect sampling strategy.

b Likelihood ratio test.

c Multivariable model *a priori* including gender, community, age category as well as demographic/behavioural factors which were associated with case/control status (i.e. whether CHiP was known to participant prior to PopART to home‐visit, whether HIV status has been disclosed, whether partner is HIV positive and on ART, and lifetime number of sexual partners).

There were very few responses in the “strongly agree” and “agree” categories for this question, responses are therefore grouped as shown to be more meaningful/increase power.

d *p*‐value for test for trend.

As was seen with “not feeling ready” to link to care (Table 2), “not feeling ready” to take ART was also associated with being a case (aOR: 2.25, 95% CI: 1.58 to 3.21, *p* < 0.001). The idea of taking life‐long treatment also appeared to be a deterrent to TLA (aOR: 1.44, 95% CI: 1.00 to 2.07, *p* = 0.05) (Table 3).

Stigmatizing attitudes were not generally seen to affect TLA (Table 3 and other data not shown). However, the more strongly participants agreed with a statement about feeling ashamed because they were HIV‐positive, the more likely they were to be a case (test for trend *p* = 0.007).

### 3.4 Differences in association by gender and country

There was no statistical evidence of differences in association by country but shown in Table S1 are associations which were seen to differ by gender. While there was no association between education of participants overall and of women alone with case/control status, men who had secondary education appeared more likely to be cases than men with primary education (aOR: 2.50, 95% CI: 1.30 to 4.82, *p* = 0.02). However, there was no clear trend seen across educational strata. Amongst men but not amongst women, being familiar with the CHiP prior to the initial PopART home visit was strongly associated with TLA (aOR of being a case among men: 0.29, 95% CI: 0.14 to 0.58, *p* < 0.001).

The majority of participants irrespective of gender or case/control status had disclosed their HIV status to someone. Among women, there was statistical evidence of disclosure encouraging TLA (aOR of being a case: 0.18, 95% CI: 0.09 to 0.36, *p* < 0.001) while the association was weak among men. Men who had a high AUDIT (Alcohol Use Disorders Identification Test) score (≥8) which denoted hazardous and harmful alcohol use 16, and possible dependence, were more likely to be cases (aOR: 2.13, 95% CI: 1.20 to 3.81, *p* = 0.009) than men with lower scores (≤7), while this was not the case among women who drank excessively.

## 4 Discussion

This study examined factors associated with the timely initiation of ART in high HIV prevalence settings in sub‐Saharan Africa, in greater detail than any other published study we are aware of. It provides novel insights into TLA in the context of UTT, with participants identified with HIV during universal door‐to‐door home‐based HIV testing. We have examined the combined outcome of TLA which requires linkage‐to‐care following HIV detection and referral in the community, and initiation of ART in the health facility, all within a relatively short period (six months). The predictors of TLA we have examined may have influenced either linkage to care or ART initiation or both. Only one other study we are aware of has examined factors associated with “linkage‐to‐ART” 17. Other studies have focused instead either on predictors of linkage‐to‐care or on predictors of initiation of ART among those already linked‐to‐care or diagnosed in health facilities. Yet, as community‐based HIV testing becomes widespread in high HIV prevalence settings, combined with immediate eligibility for ART, we consider that it is pertinent to examine TLA to effect meaningful change in ART coverage with a view to achieving the “second 90” of the UNAIDS 90‐90‐90 targets 18.

Our study found no evidence of associations between demographic or socio‐economic characteristics and TLA, similar to reported findings from other settings including one which was in the context of UTT 8, 11, 12, 14. This is encouraging for a “universal” intervention that seeks to ensure high coverage across the whole of the community. There are emerging data however which show that men and young people are less likely to initiate treatment even when diagnosed with HIV 7, 19. The greater challenge appears to be the difficulty contacting men and young people but once encountered, these groups are not more likely to decline/defer treatment initiation. Our study was not designed to examine gender differences and we observed no association between age and TLA. We focused on those who had been seen and referred for treatment by the CHiPs and this may explain the lack of association of age with TLA in our study population.

Having favourable views about the PopART CHiPs appears to facilitate TLA with prior familiarity with the CHiPs (as fellow community members), feeling comfortable talking to the CHiPs and accepting their advice, all encouraging TLA. Few participants complained of healthcare worker factors as barriers to linking‐to‐care. Clinic infrastructure factors however were cited as disincentives (inconvenient clinic opening hours, over‐crowding, etc.). This is consistent with other research which has shown that similar factors were associated with increased loss‐to‐follow up among those who have engaged with care 20.

While the evidence of associations was not strong we found that behavioural factors, including self‐reported sexual behaviour and HIV‐related behaviour (namely disclosure of HIV status) influenced TLA. Those reporting a greater number of lifetime sexual partners were more likely to succeed with TLA. This suggests that the self‐acknowledged perception that one might be at risk of HIV, facilitated linkage and ART initiation. One possible explanation is that these individuals were more willing to acknowledge living with HIV and hence achieved TLA more readily. Consistent with this explanation is the finding that those who reported “not feeling ready” or who said they would only start treatment once they felt sick, were less likely to achieve TLA. The finding that those with higher risk behaviour are more actively engaging with UTT is encouraging for treatment as prevention. The perception that treatment is not indicated until someone feels unwell is important given the persistent late presentation of patients for HIV care 21. This is an important consideration to address in future health promotion messaging.

Corroborating the findings of the study by Boyer *et al*. on ART initiation (among those linked‐to‐care) in another UTT trial, we found that disclosure of HIV‐status facilitated TLA 14. Along a similar theme, participants who reported that they had an HIV‐positive partner on ART were more likely to succeed with TLA themselves, while the opposite was true if they had an HIV‐positive partner who was not ART. Our data also indicate that participants who reported that they had disclosed their HIV‐positive status were more likely to report a partner on ART than those who had not disclosed (36% vs. 17%, respectively *p* < 0.001). Protecting a partner from acquiring HIV as a reason to start ART was not associated with increased TLA (Table 3), and this may suggest that greater efforts to promote a “treatment for prevention” message are indicated.

The high proportion of men overall who had high AUDIT scores is alarming (51%), although the prevalence in women (20%) is also substantial. The difference in association of alcohol excess with TLA by gender is interesting. Men with high AUDIT scores were more likely to be cases, and this suggests that provision of integrated alcohol reduction/treatment and HIV prevention/treatment programmes that target men should be explored. Among women, the evidence was of a weak association in the opposite direction. A possible explanation is that men with heavy alcohol consumption avoided engagement with treatment and care, while women with equivalent alcohol intake may have sought health‐care/treatment as a way to compensate for “unhealthy” behaviour. Furthermore, among women (but not men) a higher proportion of those who were heavy drinkers considered themselves at high risk of acquiring HIV infection compared to those who were non‐heavy drinkers (59% vs. 42%, *p* = 0.005). This association among women is consistent with our finding above that high risk perception may encourage treatment engagement.

Our data indicate that stigma overall is not a major barrier to TLA, but internalized stigma (i.e. taking on and believing to be true, the negative beliefs and attitudes about PLWH) may have an influence. This is said to be one of the most insidious aspects of stigma, as it somehow makes PLWH feel “less human” 22. There is evidence to suggest that support groups can help mitigate this form of stigma 23, 24.

Our study had several limitations. Only participants who agreed for the study research team to contact them could be recruited into the study. This means that individuals who could not be contacted, or declined or avoided contact would not be represented in the study, nor would those who declined to consent once contacted. While this is common to many community‐based survey studies, the representativeness of the study sample must be borne in mind as a limitation of the study. We did see evidence of differential selection of potential participants by case/control status with a higher proportion of randomly selected potential controls recruited than randomly selected potential cases (46% vs. 37%). As described earlier there were also fewer men than women in the study sample despite frequency matching on gender when randomly selecting the sample. Young people were also somewhat under‐represented in the final study sample. Both of these are likely due to the fact that the study was conducted in the household and men and younger people are less likely to be found at home than older people. Reporting bias and social desirability bias are both possibilities, in common with most research using self‐reported data. However, given the wide range of themes explored by the questionnaire and the lack of an obvious single hypothesis, these biases are unlikely to be differential based on case or control status of the participant. Reverse causality must also be borne in mind given that the CC study was conducted a period of time after the referral for HIV care. Further, given the CC design causality cannot be inferred from our findings and we are limited to observing associations.

The strengths of this study have already been alluded to, including the breadth and depth of the themes explored, the novelty of examining TLA under UTT conditions and the focus on TLA as a combination of both timely linkage‐to‐care and ART initiation, given that community‐based testing is becoming increasingly prevalent. In addition, we had a sizeable proportion of men (approximately 40%) who are often under‐represented. Our study was conducted in large urban and peri‐urban communities and this provides a good basis for generalizability for the majority of those living with HIV in sub‐Saharan Africa.

Further research is required to explain some of the results. For instance, even though familiarity with the CHiPs encouraged TLA, there is a suggestion in our data that having an HIV test with the CHiP was associated with being a case, that is, failure to achieve TLA. This is most likely due to the fact that those who tested with CHiPs were the ones receiving new diagnoses of HIV infection, and needing time to become comfortable with the idea of needing treatment, whereas those who did not test with CHiPs were the ones who already knew their HIV‐positive status and may have been more ready for TLA. However, this warrants further enquiry. Emerging evidence on the advantages of same‐day initiation of ART upon presentation at the clinic as a potential facilitator of TLA is a theme which we did not explore, but this is an important topic for future research 25.

## 5 Conclusions

In summary, our study findings suggest that the universal treatment intervention within PopART did not systematically exclude any subsets of the population and it has the potential to be universally acceptable. The finding that those who reported having an HIV‐positive partner not on ART were less likely to achieve TLA points to the cumulative gains to be won by initiating individuals on ART as it may have the added benefit that their partner would be encouraged to do so as well. As holding a favourable view of the CHiPs was associated with successful TLA, we recommend investment in the cadre of staff delivering services as a means to increase uptake. The concerns expressed by participants about health‐facilities warrants both on‐going improvement of infrastructure and innovative means to reduce the burden on clinics by providing care in the community for patients who are stable and comfortable with non‐clinic based care.

The findings that higher risk sexual behaviour may be associated with more TLA holds great promise for the effectiveness of UTT in achieving reductions in HIV‐incidence at the community level. Overall, we found few fundamental differences between those who linked‐to‐ART in a timely fashion and those who did not, but the differences we did uncover provide opportunities to achieve universal treatment coverage within the framework of UTT.

## Competing interests

We have no conflicts of interests to declare.

## Authors’ contributions

KS with oversight from RH, led on study design, writing the first draft and revisions of the paper, and performed statistical analysis of the data. CMM, CM and GH were involved with leading field data collection and ensuring the quality of data. Ab Schaap led on programming of study tools and had over‐sight of the random selection of participants and data management. Anne Stangl provided expert advice to the stigma related content of the study. Sian Floyd provided advice on statistical methods and along with Sarah Fidler and HA, provided guidance on study design and implementation. All authors contributed to the writing of the paper and have read and approved the final manuscript.

## Funding

HPTN 071 is sponsored by the National Institute of Allergy and Infectious Diseases (NIAID) under Cooperative Agreements UM1‐AI068619, UM1‐AI068617, and UM1‐AI068613, with funding from the U.S. President's Emergency Plan for AIDS Relief (PEPFAR). Additional funding is provided by the International Initiative for Impact Evaluation (3ie) with support from the Bill & Melinda Gates Foundation, as well as by NIAID, the National Institute on Drug Abuse (NIDA) and the National Institute of Mental Health (NIMH), all part of NIH. The content is solely the responsibility of the authors and does not necessarily represent the official views of the NIAID, NIMH, NIDA, PEPFAR, 3ie, or the Bill & Melinda Gates Foundation.

## Supporting information


**Table S1.** Factors with effect modification by gender, of association with case/control status

## References

[1] Cohen MS , Chen YQ , McCauley M , Gamble T , Hosseinipour MC , Kumarasamy N , et al. Prevention of HIV‐1 infection with early antiretroviral therapy. N Engl J Med. 2011;365(6):493–505. 21767103 10.1056/NEJMoa1105243 PMC3200068

[2] Granich RM , Gilks CF , Dye C , De Cock KM , Williams BG . Universal voluntary HIV testing with immediate antiretroviral therapy as a strategy for elimination of HIV transmission: a mathematical model. Lancet. 2009;373(9657):48–57. 19038438 10.1016/S0140-6736(08)61697-9

[3] INSIGHT START Study Group . Initiation of antiretroviral therapy in early asymptomatic HIV infection. N Engl J Med. 2015;373(9):795–807. 26192873 10.1056/NEJMoa1506816 PMC4569751

[4] TEMPRANO ANRS Study Group . A trial of early antiretrovirals and isoniazid preventive therapy in Africa. N Engl J Med. 2015;373(9):808–22. 26193126 10.1056/NEJMoa1507198

[5] World Health Organization . Guideline on when to start antiretroviral therapy and on pre‐exposure prophylaxis for HIV. 2015 [cited 2016 Feb 26] Available from: http://appswhoint/iris/bitstream/10665/186275/1/9789241509565_engpdf?ua=1 26598776

[6] Hayes R , Ayles H , Beyers N , Sabapathy K , Floyd S , Shanaube K , et al. HPTN 071 (PopART): rationale and design of a cluster‐randomised trial of the population impact of an HIV combination prevention intervention including universal testing and treatment ‐ a study protocol for a cluster randomised trial. Trials. 2014;15:57. 24524229 10.1186/1745-6215-15-57 PMC3929317

[7] Hayes R , Floyd S , Schaap A , Shanaube K , Bock P , Sabapathy K , et al. A universal testing and treatment intervention to improve HIV control: one‐year results from intervention communities in Zambia in the HPTN 071 (PopART) cluster‐randomised trial. PLoS Med. 2017;14(5):e1002292. 28464041 10.1371/journal.pmed.1002292 PMC5412988

[8] Aliyu MH , Blevins M , Parrish DD , Megazzini KM , Gebi UI , Muhammad MY , et al. Risk factors for delayed initiation of combination antiretroviral therapy in rural north central Nigeria. J Acquir Immune Defic Syndr. 2014;65(2):e41–9. 23727981 10.1097/QAI.0b013e31829ceaec PMC3818360

[9] Amuron B , Namara G , Birungi J , Nabiryo C , Levin J , Grosskurth H , et al. Mortality and loss‐to‐follow‐up during the pre‐treatment period in an antiretroviral therapy programme under normal health service conditions in Uganda. BMC Public Health. 2009;9:290. 19671185 10.1186/1471-2458-9-290 PMC2734853

[10] Feldacker C , Johnson D , Hosseinipour M , Phiri S , Tweya H . Who starts? Factors associated with starting antiretroviral therapy among eligible patients in two, public HIV clinics in Lilongwe, Malawi. PLoS One. 2012;7(11):e50871. 23226413 10.1371/journal.pone.0050871 PMC3511327

[11] Fox MP , Shearer K , Maskew M , Meyer‐Rath G , Clouse K , Sanne I . Attrition through multiple stages of pre‐treatment and ART HIV care in South Africa. PLoS One. 2014;9(10):e110252. 25330087 10.1371/journal.pone.0110252 PMC4203772

[12] Katz IT , Essien T , Marinda ET , Gray GE , Bangsberg DR , Martinson NA , et al. Antiretroviral therapy refusal among newly diagnosed HIV‐infected adults. AIDS. 2011;25(17):2177–81. 21832935 10.1097/QAD.0b013e32834b6464 PMC3272300

[13] McGrath N , Glynn JR , Saul J , Kranzer K , Jahn A , Mwaungulu F , et al. What happens to ART‐eligible patients who do not start ART? Dropout between screening and ART initiation: a cohort study in Karonga, Malawi. BMC Public Health. 2010;10:601. 20939872 10.1186/1471-2458-10-601 PMC2964626

[14] Boyer S , Iwuji C , Gosset A , Protopopescu C , Okesola N , Plazy M , et al. Factors associated with antiretroviral treatment initiation amongst HIV‐positive individuals linked to care within a universal test and treat programme: early findings of the ANRS 12249 TasP trial in rural South Africa. AIDS Care. 2016;28 Suppl 3:39–51. 27421051 10.1080/09540121.2016.1164808 PMC5096681

[15] Iwuji C , Orne‐Gliemann J , Balestre E , Larmarange J , Thiebaut R , Tanser F , et al. The impact of universal test and treat on HIV incidence in a rural South African population: ANRS 12249 TasP trial, 2012‐2016. 2016 [cited 2016 Oct 19]. Available from: http://programmeaids2016org/Abstract/Abstract/10537

[16] Babor TF , Higgins‐Biddle JC , Saunders JB , Monteiro MG . The Alcohol Use Disorders Identification Test ‐ guidelines for use in primary care. 2001 [cited 2017 Feb 6]. Available from: http://appswhoint/iris/bitstream/10665/67205/1/WHO_MSD_MSB_016apdf

[17] MacPherson P , MacPherson EE , Mwale D , Bertel Squire S , Makombe SD , Corbett EL , et al. Barriers and facilitators to linkage to ART in primary care: a qualitative study of patients and providers in Blantyre, Malawi. J Int AIDS Soc. 2012;15(2):18020. 23336700 10.7448/IAS.15.2.18020 PMC3535694

[18] UNAIDS . 90‐90‐90: an ambitious treatment target to help end the AIDS epidemic. 2014 [cited 2016 Jan 11] Available from: http://wwwunaidsorg/sites/default/files/media_asset/90-90-90_en_0pdf

[19] Petersen M , Balzer L , Kwarsiima D , Sang N , Chamie G , Ayieko J , et al. Association of implementation of a universal testing and treatment intervention with HIV diagnosis, receipt of antiretroviral therapy, and viral suppression in East Africa. JAMA. 2017;317(21):2196–206. 28586888 10.1001/jama.2017.5705 PMC5734234

[20] Rachlis B , Bakoyannis G , Easterbrook P , Genberg B , Braithwaite RS , Cohen CR , et al. Facility‐level factors influencing retention of patients in HIV care in East Africa. PLoS One. 2016;11(8):e0159994. 27509182 10.1371/journal.pone.0159994 PMC4980048

[21] Siedner MJ , Ng CK , Bassett IV , Katz IT , Bangsberg DR , Tsai AC . Trends in CD4 count at presentation to care and treatment initiation in sub‐Saharan Africa, 2002‐2013: a meta‐analysis. Clin Infect Dis. 2015;60(7):1120–7. 25516189 10.1093/cid/ciu1137 PMC4366582

[22] Goffman E . Stigma: notes on the management of spoiled identity. London: Penguin; 1963.

[23] Rao D , Desmond M , Andrasik M , Rasberry T , Lambert N , Cohn SE , et al. Feasibility, acceptability, and preliminary efficacy of the unity workshop: an internalized stigma reduction intervention for African American women living with HIV. AIDS Patient Care STDS. 2012;26(10):614–20. 22984780 10.1089/apc.2012.0106 PMC3462391

[24] Stangl AL , Lloyd JK , Brady LM , Holland CE , Baral S . A systematic review of interventions to reduce HIV‐related stigma and discrimination from 2002 to 2013: how far have we come? J Int AIDS Soc. 2013;16 3 Suppl 2:18734. 24242268 10.7448/IAS.16.3.18734 PMC3833106

[25] Koenig SP , Dorvil N , Devieux JG , Hedt‐Gauthier BL , Riviere C , Faustin M , et al. Same‐day HIV testing with initiation of antiretroviral therapy versus standard care for persons living with HIV: a randomized unblinded trial. PLoS Med. 2017;14(7):e1002357. 28742880 10.1371/journal.pmed.1002357 PMC5526526

