# Supplementary material for: Predictors of timely linkage‐to‐ART within universal test and treat in the HPTN 071 (PopART) trial in Zambia and South Africa: findings from a nested case‐control study
Source: J Int AIDS Soc. 2017 Dec 18;20(4):e25037. doi: 10.1002/jia2.25037 (PMC5810326; doi:10.1002/jia2.25037)
Supplement: Supplementary file 1 — Table S1. Factors with effect modification by gender, of association with case/control status [file JIA2-20-e25037-s001.docx]

**Supplementary Table 1: Factors with effect modification by gender, of association with case/control status**

|  | **Controls (Achieved TLA) N (%)** | **Cases (Did not achieve TLA) N (%)** | **Odds Ratio ^1^** | ***LRT ^2^ p-value*, 95 % Confidence Interval** | **Adjusted Odds Ratio^3^** | ***LRT ^2^ p-value,*  95 % Confidence Interval** | **Controls (Acceptors) N (%)** | **Cases (Non-acceptors) N (%)** | **Odds Ratio ^1^** | ***LRT ^2^ p-value*, 95 % Confidence Interval** | **Adjusted Odds Ratio^3^** | ***LRT ^2^ p-value,*  95 % Confidence Interval** |
| --- | --- | --- | --- | --- | --- | --- | --- | --- | --- | --- | --- | --- |
|  | **Men** | | | | | | **Women** | | | | | |
| ***Demographic, sexual behaviour and health related characteristics of cases and controls*** | | | | | | | | | | | | |
| **Educational attainment** | | | | | | | | | | | | ***p_em_ ^4^ 0.005*** |
| Primary (Grade 0-7) | 70 (46) | 45 (34) | **1** | ***0.06*** | **1** | ***0.02*** | 113 (51) | 108 (54) | 1 | *0.30* | 1 | *0.21* |
| Junior secondary (Grade 8-9) | 36 (24) | 45 (34) | **2.04** | **1.13-3.67** | **2.50** | **1.30-4.82** | 58 (26) | 39 (19) | 0.74 | 0.45-1.23 | 0.71 | 0.41-1.24 |
| Senior secondary (Grade 10-12) or higher education | 45 (30) | 42 (32) | 1.41 | 0.79-2.52 | 1.81 | 0.94-3.50 | 49 (22) | 54 (27) | 1.16 | 0.68-1.97 | 1.21 | 0.68-2.17 |
| **Was the CHiP known to the participant prior to offer of HBT?** | | | | | | | | | | | | ***p_em_ ^4^ 0.006*** |
| N | 106 (70) | 112 (85) | **1** | ***0.006*** | **1** | ***<0.001*** | 153 (69) | 141 (70) | 1 | *0.68* | 1 | *0.38* |
| Y | 45 (30) | 20 (15) | **0.41** | **0.22-0.77** | **0.29** | **0.14-0.58** | 68 (31) | 60 (30) | 0.90 | 0.57-1.44 | 0.80 | 0.49-1.32 |
| **Have you disclosed your HIV status to anyone?** | | | | | | | | | | | | ***p_em_ ^4^ 0.002*** |
| N | 24 (16) | 27 (20) | 1 | *0.27* | 1 | *0.35* | 14 (6) | 47 (23) | **1** | ***<0.001*** | **1** | ***<0.001*** |
| Y | 127 (84) | 105 (78) | 0.70 | 0.38-1.31 | 0.72 | 0.37-1.43 | 207 (94) | 154 (77) | **0.20** | **0.11-0.39** | **0.18** | **0.09-0.36** |
| **AUDIT Score** | | | | | | | | | | | | ***p_em_ ^4^ 0.02*** |
| Audit Score 7 or lower | 82 (54) | 58 (44) | **1** | ***0.05*** | **1** | ***0.009*** | 171 (77) | 168 (84) | 1 | *0.07* | 1 | *0.08* |
| Audit Score 8 or higher (hazardous and harmful alcohol use; possible dependence) | 69 (46) | 74 (56) | **1.64** | **1.00-2.70** | **2.13** | **1.20-3.81** | 50 (23) | 33 (16) | 0.63 | 0.38-1.04 | 0.61 | 0.35-1.06 |
| ***Participants perceptions of HIV service factors which may affect initiation of timely treatment*** | | | | | | | | | | | | |
| **Time constraints affecting linkage to care** | | | | | | | | | | | | ***p_em_ ^4^ 0.03*** |
| Already in care/ time not a constraint for LTC | 96 (64) | 82 (62) | 1 | *0.91* | 1 | *0.86* | 193 (87) | 149 (74) | **1** | ***<0.001*** | **1** | ***0.001*** |
| Time constraints due to livelihood/housework or both | 54 (36) | 50 (38) | 1.02 | 0.63-1.69 | 1.05 | 0.61-1.81 | 28 (13) | 52 (26) | **2.43** | **1.44-4.10** | **2.47** | **1.41-4.34** |
| ***Participants’ perceptions of advantages and disadvantages of initiation of timely treatment*** | | | | | | | | | | | | |
| **I have felt ashamed because of my HIV status** | | | | | | | | | | | | ***p_em_ ^4^ 0.02*** |
| Strongly disagree | 47 (31) | 30 (23) | 1 | *0.002* | 1 | *<0.001* | 71 (32) | 56 (28) | 1 | *0.22* | 1 | *0.20* |
| Disagree | 61 (40) | 44 (33) | 1.48 | 0.75-2.91 | 2.03 | 0.93-4.42 | 78 (35) | 62 (31) | 0.96 | 0.58-1.61 | 1.02 | 0.58-1.77 |
| Agree | 17 (11) | 35 (27) | 4.84 | 2.06-11.39 | 6.57 | 2.59-16.67 | 42 (19) | 39 (19) | 1.08 | 0.58-2.02 | 0.98 | 0.50-1.91 |
| Strongly agree | 26 (17) | 23 (17) | 1.93 | 0.87-4.25 | 2.05 | 0.86-4.86 | 30 (14) | 44 (22) | 1.72 | 0.94-3.14 | 1.81 | 0.96-3.41 |
| **People sometimes talk badly about me because I am living with HIV** | | | | | | | | | | | | ***p_em_ ^4^ 0.02*** |
| Strongly disagree | 27 (18) | 33 (25) | 1 | *0.10* | 1 | *0.16* | 56 (25) | 45 (22) | 1 | *0.19* | 1 | *0.17* |
| Disagree | 67 (44) | 58 (44) | 0.67 | 0.34-130 | 0.78 | 0.37-1.64 | 74 (33) | 86 (43) | 1.39 | 0.82-2.35 | 1.77 | 0.99-3.17 |
| Agree | 36 (24) | 33 (25) | 0.70 | 0.33-1.49 | 0.72 | 0.32-1.61 | 63 (29) | 46 (23) | 0.82 | 0.44-1.51 | 1.11 | 0.57-2.14 |
| Strongly agree | 21 (14) | 8 (6) | 0.28 | 0.10-3.05 | 0.30 | 0.10-0.89 | 28 (13) | 24 (12) | 0.93 | 0.46-1.86 | 1.31 | 0.62-2.75 |

1. A priori adjusted for gender and community to reflect sampling strategy
2. Likelihood ratio test
3. Multivariable model a priori including gender, community, age category as well as demographic/behavioural factors which were associated with case/control status (ie whether CHiP was known to participant prior to PopART to home-visit, whether HIV status has been disclosed, whether partner is HIV positive and on ART, and lifetime number of sexual partners)
4. P value for effect modification by gender
